# The Hospital Problem: Weighing and Venturing

**Published:** 1911-04-15

**Authors:** 


### The Hospital

A Journal of

The Medical Sciences and Hospital Administration.

New Series. No. 219, Vol. IX. [No. 1291, Vol. L.] SATURDAY, APRIL 15, 1911

### The Hospital Problem: Weighing and Venturing.

The psychological study of the subject of indecision is attractive to those who find a pleasure in the consideration of abstract processes. It is instructive to those who desire to learn from example, and who have the courage to accept as an axiom the statement that history gives an impartial verdict. The incalculable harm that the human race has suffered through the undecided and vacillating policy of those whom it entrusted with its social, political, and intellectual leadership is a theme for the combined talent of a philosopher and an expert jurisprudent—and even then their reflections would need the sarcastic pen of a Swift or a Voltaire to do adequate justice to the immensity of the evil engendered by such indecision. Whoever takes delight in such speculation will find plenty of material for the indictment in the study of our own social-political history. At the present time such a survey is not without its material benefit, for we are in a state of indecision with regard to our policy in reference to one of the most important social-political problems that can await the consideration of any community. We are in the transition stage between conception and realisation, either of a creation that will be permanent and valuable, or of an establishment that will, if precedents go for anything, be temporary and useless. It is possible that these two terms, creation and establishment, do not sound at all impressive or convincing, but a momentary reflection will show that they are deliberately chosen to exemplify the possibilities that confront us.

For centuries we have had in this country a hospital system which has proved itself to be adequate to our needs, which has been a source of moral strength to us, and of material benefit to millions of sick people whose relief from distress and suffering was made possible by the voluntary effort of millions of their fellow beings. This system has not stagnated with age; through it has run a continual current of progress, which has enabled it to absorb and profit by every known evolution in medical science and every advance that was an improvement. No fair presentation of the case for or against the Voluntary System can abrogate a tithe of the merit that this system possesses as a factor in the moral and intellectual development of the nation. The arguments advanced against it are not so much derived from its own impotence to deal with new conditions and an altered state of things as from the alleged conflict between it and certain factors which this new condition of affairs has brought into being. The overlapping of which we hear so much, and which no one who has studied the matter would care to deny, is not an argument against the existence of the Voluntary System; it is merely a proof that there exists less co-operation between, and a diminished control of, the voluntary hospitals and the State-aided institutions for the relief of the sick and indigent. It would be foolish to complain that a system has lost its right of being because its utility has been, to a certain extent, rendered supererogatory by the establishment of a subsidiary system. The fact that it has been overlapped only tends to show that both systems are in urgent want of readjustment.

"Erst wägen dann wagen!" the philosophic motto of the daring Moltke, is here as much applicable as it is to military tactics. We have weighed and considered for many years, and our matured reflections have convinced us that certain reforms are necessary, desirable, and expedient. The labours of the Commissioners on the Reform of the Poor Law have given us the various sums total of the considerations that go to prove this premiss. In the diversity of these considerations lies not a danger to the ultimate realisation of the ideal, but a hope for the maintenance of that public interest in reform which alone can render combined action, no matter what ultimate shape it may take, possible. We are far from regretting such diversity of opinion. In the present state of public knowledge—or rather in the present state of public ignorance—nothing would be more lamentable or less hopeful than a consensus of opinion on the important problems that confront us in the domain of institutional reform. Such solidarity of thought, such unanimity in the verdict, would give us reason to pause and to ask ourselves whether it was the outcome of real, innate conviction, or merely the expression of impatience which leaps at the first suggestion and clings with stubborn obstinacy to a ready-made decision which is supported by the influence of a majority.

We venture to think that no one interested in hospital reform and who has seriously reflected on the subject, will need any assistance in combating the preconceived notion that the voluntary hospital system is on its last legs. That being the case it is justifiable to ask what prevents us from co-operating in its defence and reorganisation. We have earnestly and conscientiously weighed; it is now the time to act. We assert that the time has come to attempt to draw certain definite conclusions from our extended considerations, to join together in attempting a solution of the difficulties that prevent unanimous action, and to find a starting point. That, in fact, is the essential in co-operation, and it is to co-operation that we must look for a settlement of the difficulties. Co-operation has meant much to the system of State insurance in the German Empire; it is no exaggeration to say that without it that system could never have attained the success which it undoubtedly has. It has meant equally much for the hospital system in Switzerland and Holland; it is likely to mean as much for the hospitals in our overseas dominions. When we have these examples before us, is it unreasonable or arrogant to demand that we should at least give co-operation amongst all agencies dealing with medical relief a fair trial in this country before we accept as proven the existence of a dilemma which implies an amount of social and institutional degeneration on which no country, however conceited, could afford to pride itself?

